# Magnetocardiography to screen adults with arrhythmogenic cardiomyopathy: A feasibility study

**DOI:** 10.1016/j.ahjo.2026.100813

**Published:** 2026-06-10

**Authors:** Samuel Friese, Philipp Wunderl, Tobias Jensch, Lena Wunderl, Cordula M. Wolf, Bettina Reich, Christian Meierhofer, Reinhard Heckel, Isabel Diebold, Eimo Martens, Dominik Westphal, Peter Fierlinger, Peter Ewert, Annette Wacker-Gussmann

**Affiliations:** a Chair for Precision Measurements at Extreme Conditions, Physics Department, TUM School of Natural Sciences, Technical University of Munich, Garching, Germany; b Department of Computer Engineering, TUM School of Computation, Information and Technology, Technical University of Munich, Munich, Germany; c Department of Human Genetics, Landeskrankenhaus University Hospital, Paracelsus Medical University, Salzburg, Austria; d Clinic of Congenital Heart Disease and Pediatric Cardiology, German Heart Center, TUM University Hospital, Technical University of Munich, Munich, Germany; e Department of Internal Medicine I, TUM University Hospital, Klinikum rechts der Isar, TUM School of Medicine and Health, Technical University Munich, Germany

**Keywords:** Magnetocardiography, Cardiomyopathy, Magnetic field, Screening, Innovative technology, ACM

## Abstract

**Background:**

Arrhythmogenic cardiomyopathy (ACM) is a primary cardiomyopathy associated with mechanical and electrical dysfunction, making diagnosis a clinical challenge. This feasibility study investigates whether Optically Pumped Magnetometer (OPM)-based magnetocardiography (MCG), which offers complementary spatial information about cardiac electrical activity compared to ECG, can identify biomarkers in ACM patients.

**Methods:**

MCG data were acquired using an array of 16 OPMs. After signal processing and artifact removal via Independent Component Analysis (ICA), two-dimensional vector magnetocardiogram (VMCG) projections were constructed for the QRS and T-wave segments. Geometric features derived from the VMCG loops were compared between groups using Welch's *t*-test. To ensure statistical robustness, Monte Carlo simulations modeled measurement uncertainty, correlation analyses assessed potential age-related confounding, and post-hoc false discovery rate (FDR) correction was applied to account for repeated sensor-wise comparisons.

**Results:**

We enrolled 9 ACM patients and 12 healthy controls. The main finding was signal attenuation in ACM patients compared to healthy controls, as evidenced by smaller VMCG loop dimensions. This effect was most pronounced during the T-wave segment, where ACM patients exhibited a smaller convex hull area. During the QRS complex, reductions in maximum signal amplitude were observed. Correlation analysis in healthy controls revealed no significant association between age and signal strength. No significant differences were found in the ST segment.

**Conclusion:**

Our findings suggest that flexible, room-temperature OPM-based MCG may non-invasively detect electrophysiological alterations in ACM affecting both ventricular repolarization (T-wave area) and depolarization (QRS amplitude), with repolarization changes being particularly prominent. Further evaluation in larger studies is warranted.

## Highlights

• OPM-MCG offers a flexible, room-temperature alternative to SQUID systems.
• MCG detects signal attenuation in Arrhythmogenic Cardiomyopathy (ACM).
• Reduced T-wave Convex Hull Area serves as a robust ACM biomarker.
• Signal attenuation correlates with disease, not patient age.
• OPM-MCG shows potential for non-invasive screening of at-risk adults.

## Graphical abstract

**Figure:**
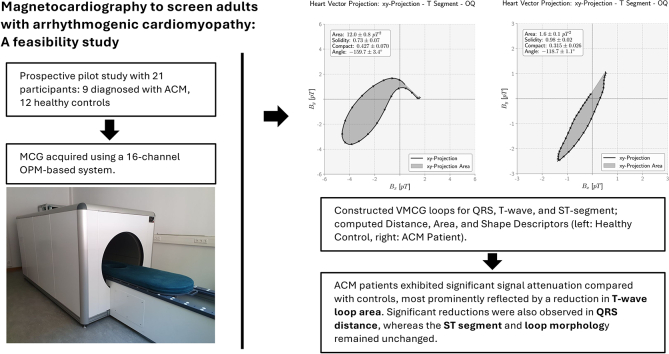

## 1 Introduction

The World Health Organization (WHO) defines primary cardiomyopathy as “cardiomyopathy associated with a mechanical or electrical dysfunction of the heart”. According to the ESC guidelines 2022 [1], one of these cardiomyopathies, which includes both dysfunctions, is arrhythmogenic cardiomyopathy (ACM, previously ARVC). ACM preferentially affects the right ventricle (but also the left ventricle), in which heart muscle cells are replaced by fibro-fatty tissue during the disease. This ventricular remodeling leads to arrhythmogenicity, which gives the disease its name [2]. The prevalence is estimated to be between 1:1000 and 1:1250 [2], making it one of the more common rare diseases. The onset of the disease in adults is, on average, 31 years, but it can vary considerably (4–64 years) [2]. Clinical criteria have been established to determine the probability of ACM (so-called task force criteria). These criteria, revised in 2010 and 2020 (“The Padua criteria”), include family history, abnormalities on imaging (echocardiography, CMR), and rhythm analyses (resting and long-term ECG). The clinical diagnosis of ACM is considered confirmed if two major criteria, one major and two minor criteria, or four minor criteria are present [3].

A major criterion is detecting a disease-causing variant in one of the known associated disease genes. These disease genes often encode desmosomal proteins that mediate cell-cell interactions. Variants are most frequently found in the PKP2, DSP, and DSG2 genes [2]. However, a pathogenic variant can only be detected in around 50% of patients with task force-positive ACM [4].

A phenotype-genotype correlation (statement about the course of the disease based on the detected pathogenic genetic variant) is only possible to a limited extent. Patients with a pathogenic variant in DSP are more likely to have left ventricular involvement [5]. Overall, the course of the disease can vary considerably within a family with the same pathogenic variant [6]. This makes counseling and treating patients and their family members challenging, especially when it comes to prognostic statements. This clinical problem highlights the need to develop additional clinical parameters beyond the existing ones to better characterize the disease in individual patients.

Magnetocardiography (MCG) provides a direct view of cardiac electrophysiology, complementary to the ECG, as magnetic fields permeate biological tissue without attenuation. While clinical adoption was previously hindered by the rigid, cryogenic nature of SQUID systems [7], Optically Pumped Magnetometers (OPMs) have emerged as a versatile alternative. Operating at room temperature, OPMs allow flexible sensor placement closer to the chest wall, potentially improving the detection of localized substrates. Here, we investigated the feasibility of this novel OPM technology to distinguish ACM patients from healthy controls.

## 2 Methods

### 2.1 Subjects

The study cohort included participants diagnosed with ACM who presented at our university hospital and healthy subjects. Inclusion criteria for ACM participants were a) Task force positive ACM patients with evidence of pathogenic variants (clinically and genetically confirmed ACM), or b) Task force positive ACM patients without evidence of a pathogenic variant (clinically confirmed ACM), or c) Task force negative carriers of a pathogenic variant (genetic predisposition to develop ACM).

Exclusion criteria were minors, inability to provide consent, inability to understand the study information because of a language barrier, and implanted cardiac electronic devices, including ICDs or pacemakers. Healthy controls were those with no history of cardiac disease and who were unremarkable on physical examination.

### 2.2 Magnetocardiography

Magnetocardiography (MCG) is a non-invasive imaging modality that maps the weak magnetic fields generated by cardiac electrical activity. While historically reliant on cryogenic Superconducting Quantum Interference Devices (SQUIDs), the recent advent of Optically Pumped Magnetometers (OPMs) has significantly reduced the cost and complexity of the technology, making clinical application more feasible [8].

The fundamental advantage of MCG over standard electrocardiography lies in the physical nature of the recorded signal and its interaction with the body. The ECG measures scalar surface potentials generated by passive, extracellular volume currents. These currents flow through the body and are strongly dependent on the heterogeneous electrical conductivities of the intervening tissues. High-impedance layers, such as subcutaneous fat or lung tissue, significantly attenuate and distort electrical potentials [7].

Conversely, the MCG is directly sensitive to the magnetic field generated by intracellular primary currents. Since biological tissues are magnetically transparent, the cardiac magnetic field propagates to the sensor array without the attenuation inherent to surface potentials. Crucially, this implies that observed changes in magnetic signal amplitude are a direct reflection of the underlying myocardial source strength. Consequently, multi-channel MCG arrays provide a high-fidelity spatial map of cardiac activity that complements the information resolved by the standard 12‑lead ECG [8].

### 2.3 Measurement setup and data acquisition

**Fig. 1:**
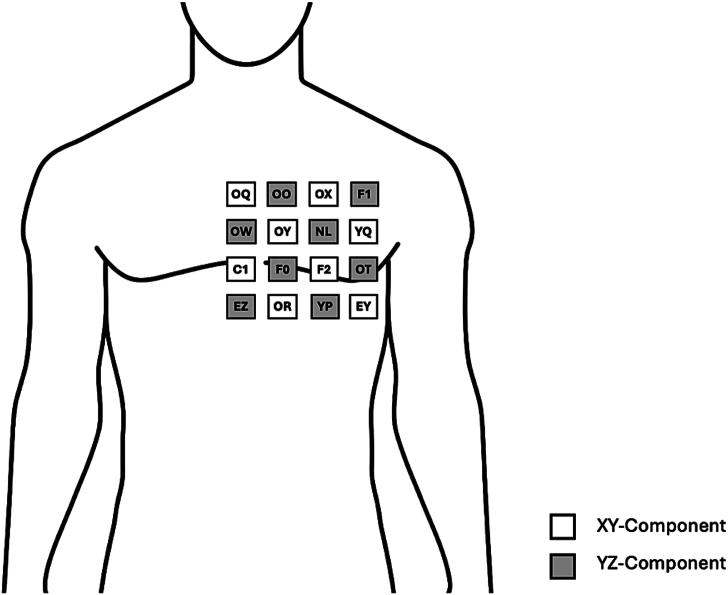
Layout of the 4 × 4 grid of dual-axis QuSpin sensors, including sensor IDs, magnetic field components measured, and position relative to the patient's torso.

For the measurements, magnetic shielding was provided by a cylindrical, person-sized enclosure composed of three layers of mu-metal to attenuate ambient magnetic noise. Additional active field compensation was implemented using the tri-axial Magnetic Field Cancelling System MR-3 (Stefan Mayer Instruments, Dinslaken, Germany), resulting in an ambient noise floor of about 80 fT/√Hz. A set of sixteen QuSpin Zero Field Magnetometers (QZFMs; QuSpin Inc., Louisville, CO, USA) was arranged in a flat 4 × 4 grid with 25.3 mm spacing and positioned beneath the subject's chest (Fig. 1). The specific array placement (lower/right region) was chosen to target the right ventricular anatomy. All OPM sensors were operated in dual-axis mode, except for one triaxial sensor.

Participants wore non-magnetic clothing. While both positions were initially considered, only data recorded in the prone position were included in the final analysis, as supine measurements demonstrated lower data quality. Each recording lasted at least 5 min. Although 21 participants were measured, sporadic sensor malfunctions necessitated the exclusion of specific channels. As a result, the number of valid observations for certain sensors fell below the total participant count (N < 21). The complete measurement procedure took approximately 10 min.

### 2.4 Signal processing and averaging

MCG data were preprocessed using a digital bandpass filter (1–95 Hz) to preserve relevant cardiac frequencies and a band-stop filter to attenuate 50 Hz powerline interference and its harmonics. Baseline drift and DC offset were removed via third-order polynomial detrending.

A lightweight neural network segmented the MCG signal into four waveform classes: no wave, P wave, QRS complex, and T wave. Based on the predicted segment durations, a heartbeat plausibility score was computed by comparing the relative segment lengths to physiologically expected ranges and averaging the neural network's confidence. This score guided artifact removal through Independent Component Analysis (ICA). Specifically, ICA was applied separately to each magnetic field component (x, y, z), using the respective sensor channels as input. Output components showing high similarity to valid heartbeat morphology, based on the plausibility score, were retained, whereas components with low cardiac relevance were discarded. This method allowed us to preserve the essential vectorial information of the cardiac signal while effectively removing noise sources.

For QRS detection, the single cleanest channel, selected from all sensors and their two orthogonal components, was used. QRS complexes were identified within this channel over intervals spanning at least 30 s. Subsequently, average waveform segments centered around the detected QRS peaks were computed using a windowed averaging approach to enhance signal-to-noise ratio and suppress statistical fluctuations.

### 2.5 Measurements

All subsequent analyses were performed on the average MCG waveforms. We assumed that, due to the proximity of the sensors to the heart, cardiac activity was recorded simultaneously across the entire sensor grid. The boundaries of the P wave, QRS complex, and T wave of the averaged waveforms were initially estimated by the neural network and subsequently refined through manual review. A relatively large timing uncertainty, modeled as a Gaussian distribution centered at 0 ms with a standard deviation of ±40 ms, was deliberately assumed for each manually adjusted segment boundary to rule out potential biases introduced by differing segmentations. Feature extraction was subsequently performed on three key cardiac intervals defined by these boundaries: the full QRS complex, the ST segment (spanning from the J-point to the onset of the T-wave), and the complete T-wave.

**Fig. 2:**
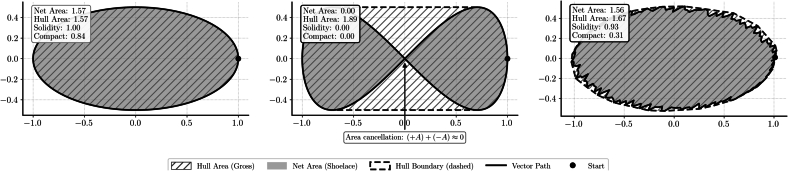
Example VMCG projections during the T-wave segment, along with calculated features for the OQ sensor (Fig. 3), for **(a)** a healthy participant and **(b)** a patient diagnosed with ACM. (Note that axes are scaled differently.

For each of the three defined segments and every sensor, we constructed a two-dimensional vector magnetocardiogram (VMCG) [8]. For each sensor, the time-varying magnetic vector B(t) was projected onto the xy and yz planes (depending on the components measured; Fig. 1), forming a trajectory loop. Unlike scalar ECG intervals, these loops capture both the spatial and temporal evolution of the depolarization and repolarization wavefronts (Fig. 2).

**Fig. 3:**
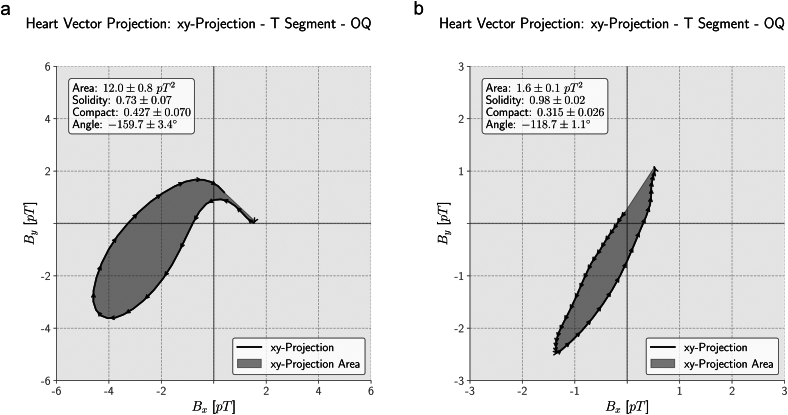
Comparison of Net Area (signed polygon; dark gray fill, black boundary) and Convex Hull Area (hatched area, dashed boundary) across three scenarios. **(a)** Standard Elliptical Shape: Represents a coherent trajectory where Net and Hull areas are identical, yielding a Solidity (Net / Hull) of ≈1.0 and high Compactness (not 1 as the shape isn't a perfect circle). **(b)** Self-Intersecting Loop: A “figure-8” trajectory where the positive and negative signed components of the Net Area mathematically cancel out (approaching 0). The Hull Area remains unaffected, capturing the full spatial extent, resulting in a distinctly low solidity and compactness score. **(c)** Fragmentation: A trajectory characterized by jagged path deviations. While the general convex shape is preserved (Solidity ≈ 1.0), the increased perimeter length significantly reduces the Compactness score (4π · Area / Perimeter^2^).

To systematically quantify the morphology of these vector loops, we extracted a set of geometric features based on the methodology recently established by Brala et al. [9]. These features were selected to characterize both the magnitude (size) and the topology (shape) of the magnetic field trajectory:

1. Maximum magnetic field amplitude (“distance”)

• Definition: The maximum Euclidean distance from the loop's origin to its furthest point.
• Physiological Significance: This proxies the peak magnitude of intracellular currents. A reduced distance implies signal attenuation, potentially due to the loss of viable myocardium (fibro-fatty replacement) characteristic of ACM.

2. Convex hull area (total spatial dispersion)

• Definition: The area of the smallest convex polygon enclosing the vector trajectory (Fig. 3, dashed boundary, hatched area).
• Physiological Significance: This feature captures the magnitude and asymmetry of the magnetic field vector over a segment (e.g., the T wave). A larger area signifies a distinct divergence between the initial and terminal phases, whereas a minimal area characterizes either signal attenuation or a symmetric return to baseline.

3. Net area (signed area)

• Definition & Significance: Calculated using the Shoelace formula, this signed metric accounts for loop directionality (Fig. 3, gray-shaded area). Unlike convex hull area, self-intersecting loops (e.g., figure-8 patterns) result in the mathematical cancellation of positive and negative areas, distinguishing open loops from complex, fragmented patterns.

4. Shape descriptors (solidity & compactness)

Two dimensionless ratios assessed morphology independent of amplitude:

• Solidity (Net Area / Hull Area): Quantifies loop coherence. Values near 1.0 indicate open loops; low values identify twisting or self-intersections (Fig. 3).
• Compactness (4*π*·*Area* / *Perimeter*^*2*^): Quantifies circularity. High values indicate regular, round shapes; low values imply elongated or irregular trajectories.

### 2.6 Statistical analysis

Statistical comparisons of magnetic heart vector features were performed between ACM subjects and healthy controls, across three cardiac segments (QRS complex, ST segment, and T wave) and the geometric features.

Group differences for each segment–metric combination were assessed using Welch's two-sample *t*-test, with two-tailed p-values < 0.05 considered statistically significant. Diagnostic performance was evaluated using receiver operating characteristic (ROC) curve analysis, with optimal thresholds determined by maximizing Youden's index. Sensitivity, specificity, F₁-score, and area under the curve (AUC) were reported for each threshold. Given the exploratory nature of this feasibility study and the limited sample size, ROC-derived thresholds were considered preliminary and were not interpreted as validated diagnostic cutoffs. To evaluate the robustness of our findings against measurement imprecision, specifically the uncertainty in defining the exact start and end times of cardiac segments, we incorporated measurement uncertainty via a Monte Carlo simulation with N = 5000 iterations. This simulation served as a sensitivity analysis to ensure that significant differences are not artifacts of specific segmentation choices. In each iteration, synthetic datasets were sampled from normal distributions centered at the nominal metric values, with standard deviations derived from the measurement uncertainty (corresponding to the modeled ±40 ms segmentation tolerance). Statistical tests and threshold determinations were repeated on these iterations to estimate 95% uncertainty intervals (UI) for the statistical parameters, including p-values. A result was considered highly robust only if the entire 95% UI for the p-value remained below the 0.05 significance level, indicating that the finding persists even when accounting for measurement error. The UI reflects the range in which the considered metric would fall in 95 out of 100 repeated experiments. To assess whether age differences between groups may have confounded the analysis, we examined the association between age and magnetic field strength within the healthy control group using Spearman's rank correlation. Because this analysis was conducted as a conservative confounder check, Bonferroni correction was applied across the sensor array, with a significance threshold of α = 0.05/16 ≈ 0.003. For each cardiac segment–feature combination in the VMCG analysis, false discovery rate (FDR) correction was applied across the 16 sensor/projection entries using the Benjamini–Hochberg procedure at q = 0.05 to account for repeated sensor-wise comparisons while preserving sensitivity to spatially distributed effects. All statistical analyses were performed in Python version 3.11.2.

## 3 Results

This prospective feasibility study enrolled 21 participants, comprising 9 patients diagnosed with ACM and 12 healthy controls. The study was conducted as basic research. The mean age of the ACM cohort was 41 (SD ± 14) years, and all diagnoses were evaluated by a referring cardiologist (Table 1). The healthy controls had a mean age of 31 (SD ± 9) years; none had a history of congenital or acquired cardiac disease or were taking any medication. The baseline characteristics of the ACM patients are provided in Table 1.

**Table 1:** Clinical, genetic, and imaging characteristics of the ACM study cohort. (Abbreviations: ACM: Arrhythmogenic Cardiomyopathy; TFC: Task Force Criteria; MRI: Magnetic Resonance Imaging; RVEF: Right Ventricular Ejection Fraction; VES: Ventricular Extrasystoles; BBB: Bundle Branch Block.)

Pts	Age	Gender	TFC criteria major	TFC criteria minor	TFC Pos	Genetic findings	MRI findings	Echo	ECG	Family history
1	53	Female	2	1	yes	c.2014-1G > C, *PKP2* (NM_001005242.2), heterozygous, pathogenic	Right ventricle (RV) dilated, dyskinesia anterolateral and right ventricle, interseptal microaneurysmata RVEF 35%	Hypo- and akinesia of the apical free wall	>2500 VES in 24 h	Neg
2	39	Male	2	0	yes	Negative	Transmural fibrotic area on the lateral wall of the right ventricle midventricular with thinning of the myocardium, motility disorder, RVEF 34%		Negative T wave in III, aVR and V1	Pos
3	46	Male	2	0	Yes	Negative	Late Gadolinium Enhancement, septal right ventricle	VES during measurements, otherwise normal	VES complete BBB, ventricular tachycardia	Pos.
4	60	Female	2	0	Yes	c.2014-1G > C, *PKP2* (NM_001005242.2), heterozygous, pathogenic	n.a.	RV Apex Aneurysm	Ventricular Tachycardia	Pos
5	21	Female	2	0	Yes	c.2014-1G > C, *PKP2* (NM_001005242.2), heterozygous, pathogenic	RV normal, EF RV 65%, no regional bulging, LV EF 71%	Dyskinesia	Sinus rhythm, without pathological findings	Pos
6	56	Female	2	0	Yes	Test pending	circumscribed hypokinesia in the midventricular region of the inferolateral wall, LVEF in the lower normal range, basal inferior fibrosis	Normal	Sinus rhythm, without pathological findings	Pos
7	30	Male	1	2	Yes	Negative	RVEF 38%, hypokinesia apical right ventricle	Normal	Late Potential positive	Pos
8	52	Female	1	2	Yes	c.369G > A, p(Trp123*), *PKP2* (NM_001005242.2), heterozygous, pathogenic	Small aneurysms on the right ventricle, especially in the right ventricular outflow tract and in the inferior right ventricular wall RV 53%	Mild RV bulging and RV dyskinesia, RV function within normal limits	Sinus rhythm, without pathological findings	Pos
9	35	Female	1	2	Yes	Mutation in DSP*	RVEF 41% late enhancement posterolateral left	Global hypo-kinesia, EF 31%	AV Block I degree, VES (<500 /24 h)	Neg

### 3.1 General VMCG characteristics

**Fig. 4:**
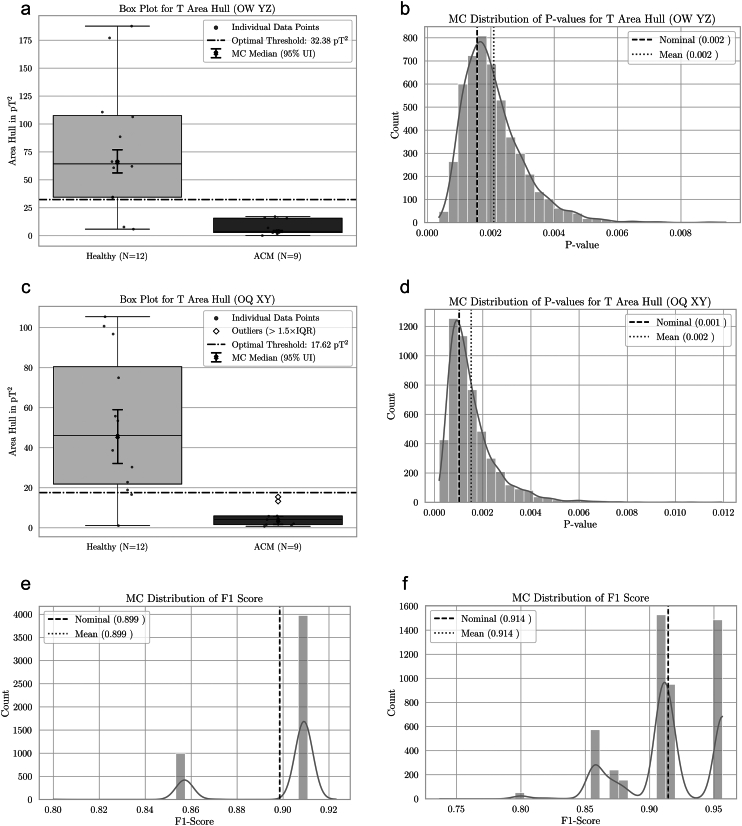
T-wave convex hull area analysis for sensors OW and OQ (Fig. 1). **(a, c)** Boxplots of nominal VMCG area values (in pT^2^). The shaded boxes represent the interquartile range (IQR), while the bold error bars denote the 95% uncertainty intervals of the median, estimated via Monte Carlo simulation. This interval reflects the range in which the true median would fall in 95 out of 100 repeated experiments. The nominal median is indicated by a horizontal line. Note that sensor OW **(a)** recorded the yz-projection and sensor OQ **(c)** recorded the xy-projection (Outliers were included in the statistical analysis). **(b, d)** Distributions of p-values from the Monte Carlo simulations for OW and OQ, respectively. **(e, f)** Distributions of F1 scores from the same simulations for OW and OQ, indicating classification performance.

**Fig. 5:**
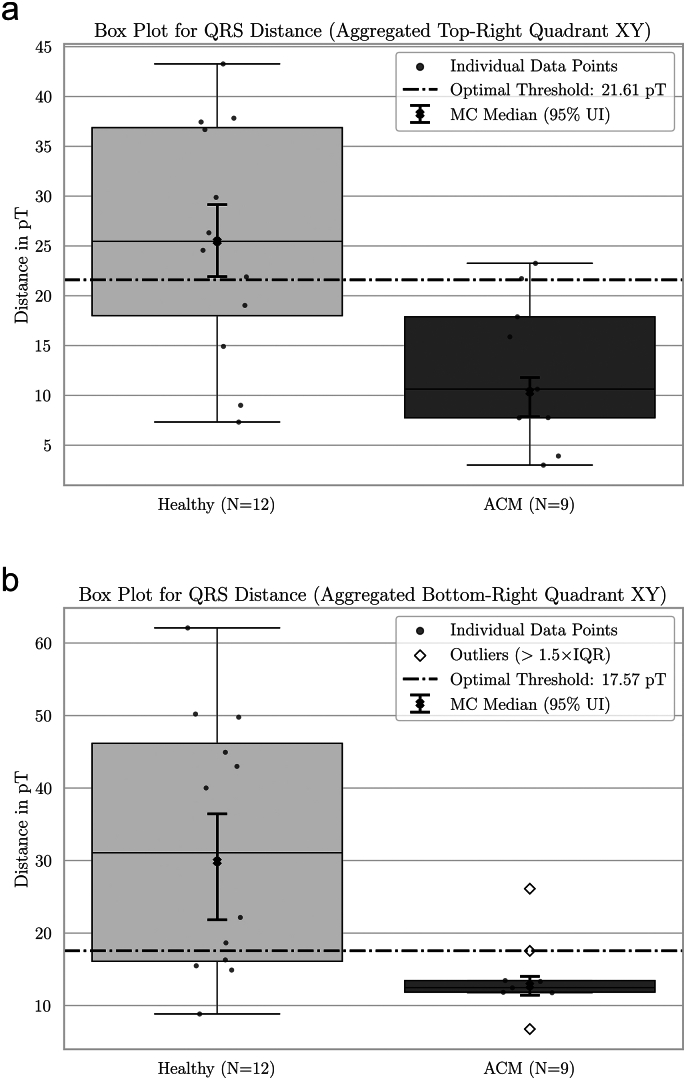
Boxplots following the same layout as Fig. 4, showing aggregated results for the xy-component of the QRS distance metric in the **(a)** top-right sensors and **(b)** bottom-right sensors.

The primary finding of this study is a statistically significant reduction in the VMCG loop dimensions in ACM patients compared to healthy controls. This was most pronounced during ventricular repolarization (T-wave) and, to a lesser extent, during depolarization (QRS complex). Specifically, ACM patients exhibited significantly lower values for convex hull area (total dispersion) and Distance (maximum amplitude) (Fig. 4, Fig. 5).

In contrast, no significant differences were found in the ST segment. Notably, the shape descriptors: Solidity (Twist Index) and Compactness, did not differ significantly between groups. This suggests that, while the ACM signal is attenuated (due to a smaller loop area and amplitude), the loop topology remains coherent and does not exhibit increased twisting or fragmentation in these 2D projections compared to the controls.

To assess age as a potential confounder for this attenuation, we analyzed the relationship between age and magnetic field strength within the healthy control group. Using Spearman's rank correlation and applying a Bonferroni correction for multiple comparisons, no significant correlation was observed between age and the T-wave convex hull area in any sensor. This suggests that the observed signal reduction is more likely disease-related than age-related in this cohort.

### 3.2 T-wave analysis

**Fig. 6:**
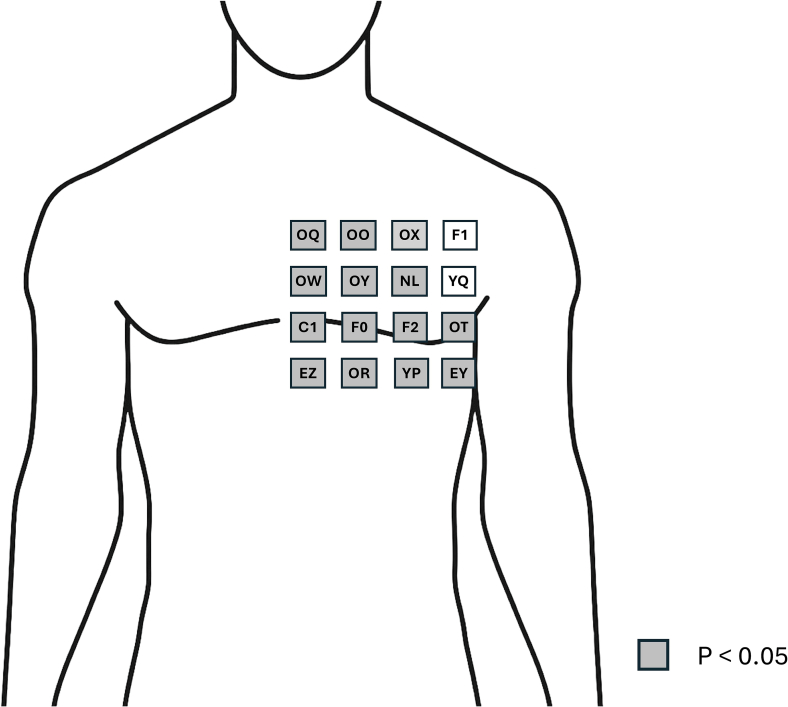
Map of the 4 × 4 sensor grid (patient's perspective) showing sensors (in gray) with an average) statistically significant difference (p < 0.05) in the T-wave VMCG convex hull area between healthy subjects and those with ACM.

Analysis of the T-wave convex hull area revealed significant differences in 14 of 16 sensor/projection entries. Spatially, these differences were concentrated in the lower and right regions of the sensor array, corresponding to the ventricular positions (Fig. 6). Crucially, for 13 of these 14 nominally significant entries, the entire 95% uncertainty interval (UI) for the p-value remained below the 0.05 threshold (Table 2), indicating that the distinction in convex hull area persisted when accounting for segmentation uncertainty. In the post-hoc FDR correction, also 13 of 16 sensor/projection entries remained significant after correction for repeated sensor-wise comparisons. The FDR-surviving effects retained the same direction, with smaller T-wave convex hull areas in ACM patients, and preserved the lower/right spatial distribution observed in the nominal analysis. A similar, though slightly less widespread, reduction was observed in the T-wave Distance metric (significant in 10 of 16 sensor/projection entries, 8 of 16 after FDR correction). Additionally, the diagnostic thresholds appeared higher in the yz projections compared to the xy projections (Fig. 4).

**Table 2:** Mean p-values and 95% uncertainty intervals for the T-wave convex hull area metric across the sensors that showed significant differences.

Sensor	Mean p-value	95% UI lower	95% UI upper
F0	0.0036	0.0010	0.0089
OR	0.0271	0.0123	0.0479
EZ	0.0110	0.0019	0.0393
OX	0.0171	0.0063	0.0361
OW	0.0021	0.0008	0.0045
OO	0.0108	0.0053	0.0190
C1	0.0015	0.0001	0.0060
OY	0.0067	0.0018	0.0174
F2	0.0196	0.0099	0.0355
EY	0.0463	0.0171	0.0917
YP	0.0142	0.0026	0.0339
NL	0.0269	0.0125	0.0491
OT	0.0106	0.0037	0.0236
OQ	0.0015	0.0004	0.0042

### 3.3 QRS complex analysis

In addition to repolarization abnormalities, our analysis revealed distinct alterations during ventricular depolarization (QRS complex). Most notably, the QRS Distance (maximum amplitude) was significantly reduced in 12 of 16 sensor/projection entries. Eight of these entries demonstrated statistical robustness (upper 95% UI < 0.05; Table 3), indicating a widespread reduction in magnetic field amplitude. After FDR correction for repeated sensor-wise comparisons, QRS Distance remained significant in 9 of 16 sensor/projection entries. The QRS convex hull area was also significantly smaller in 8 of 16 sensor/projection entries.

**Table 3:** Mean p-values and 95% uncertainty intervals for the QRS-wave distance metric across the sensors that showed significant differences.

Sensor	Mean p-value	95% UI lower	95% UI upper
F0	0.0190	0.0049	0.0484
OR	0.0106	0.0042	0.0233
F1	0.0467	0.0170	0.1017
OW	0.0063	0.0018	0.0141
OO	0.0034	0.0004	0.0124
C1	0.0108	0.0023	0.0319
OY	0.0093	0.0015	0.0287
F2	0.0016	0.0007	0.0037
YP	0.0334	0.0113	0.0783
NL	0.0011	0.0001	0.0042
YQ	0.0241	0.0058	0.0640
OQ	0.0385	0.0038	0.1451

### 3.4 Aggregated spatial analysis

To mitigate spatial uncertainty arising from anatomical variability and array positioning, we performed an aggregated sub-grid analysis. The 4 × 4 array was partitioned into four 2 × 2 quadrants, and vector components within each quadrant were averaged (e.g., combining adjacent sensors in the bottom-right corner) patient-wise. The statistical analysis was then repeated on these averaged datasets. The results are shown in Table 4, Table 5 and Fig. 5. As in the individual sensor analysis, the most significant effects were observed in the lower and right half of the grid.

**Table 4:** Mean p-values and 95% uncertainty intervals (UI) for the aggregated analysis of T-wave convex hull area across significant quadrants of the sensor array (from the patient's perspective).

Source	Mean p-value	95% UI lower	95% UI upper
Top Left (yz)	0.0195	0.0037	0.0625
Top Right (xy)	0.0023	0.0006	0.0064
Top Right (yz)	0.0042	0.0017	0.0083
Bottom Left (xy)	0.0253	0.0106	0.0496
Bottom Left (yz)	0.0103	0.0021	0.0257
Bottom Right (xy)	0.0085	0.0023	0.0207
Bottom Right (yz)	0.0015	0.0004	0.0043

**Table 5:** Mean p-values and 95% uncertainty intervals (UI) for the aggregated analysis of the QRS-complex distance metric across significant quadrants of the sensor array (from the patient's perspective).

Source	Mean p-value	95% UI lower	95% UI upper
Top Left (yz)	0.0052	0.0011	0.0146
Top Right (xy)	0.0109	0.0013	0.0397
Top Right (yz)	0.0029	0.0005	0.0092
Bottom Left (xy)	0.0183	0.0089	0.0354
Bottom Right (xy)	0.0074	0.0024	0.0185
Bottom Right (yz)	0.0366	0.0086	0.0985

#### 3.4.1 T-wave area

Aggregated analysis of the T-wave convex hull area corroborated the findings from the single-sensor analysis. Seven of eight quadrant/projection entries demonstrated significant differences and all seven remained significant after FDR correction. With the exception of the top-left (yz) component, all FDR-significant entries also demonstrated robust statistical significance after accounting for segmentation uncertainty (95% UI < 0.05), as shown in Table 4. The strongest effects were identified in the right-sided quadrants. Specifically, the bottom-right (yz) component showed the lowest mean p-value (0.0015; 95% UI: 0.0004–0.0040), followed closely by the top-right (xy) component (p = 0.0023; 95% UI: 0.0005–0.0066).

#### 3.4.2 QRS complex distance

The aggregated analysis of the QRS Distance (maximum amplitude) also revealed significant signal attenuation in ACM patients (Table 5, Fig. 5). Significant differences were identified in six of eight quadrant/projection entries, and all six remained significant after FDR correction. Robust differences after accounting for segmentation uncertainty (UI < 0.05) were identified across multiple quadrants, including the top left, top right, and bottom left. However, the top-right (yz) component exhibited the strongest effect (p = 0.0029; 95% UI: 0.0005–0.0092).

## 4 Discussion

The primary finding of this study is a significant reduction in magnetic field strength in ACM patients compared to healthy controls, as evidenced by smaller VMCG loop areas and shorter loop lengths.

This signal attenuation was most pronounced during the T-wave (repolarization), particularly for the convex hull area, but was also observed in the QRS complex. The principal T-wave convex hull area finding persisted after correction for repeated sensor-wise comparisons and remained robust against segmentation uncertainty in the Monte Carlo analysis. Notably, the intra-group correlation analysis in healthy controls supports the conclusion that the observed signal attenuation is likely disease-driven rather than age-driven, despite the lack of age-matched controls.

These results are consistent with the underlying pathophysiology of ACM, where functional cardiomyocytes are progressively replaced by fibro-fatty tissue. As the mass of viable myocardium decreases, the magnitude of the generated intracellular currents, and consequently the resulting magnetic fields, would be expected to diminish. The fact that shape factors remained stable lends support to this interpretation. It implies that although the magnitude of active myocardial sources is reduced, the spatial coherence of the remaining electrical activity appears to be largely maintained. However, it remains unclear whether the observed signal attenuation (reduced VMCG loop area) is specific to the fibro-fatty replacement in ACM or potentially a general feature of other cardiomyopathies (e.g. dilated cardiomyopathy or ischemic heart disease). Therefore, larger studies with a higher number of cases, including other cardiac pathologies, are necessary to confirm the specificity of these OPM-MCG biomarkers for ACM.

This proposed relationship between tissue loss and signal reduction is difficult to establish reliably using standard surface ECG. ECG potentials are heavily attenuated and distorted by patient-specific factors, such as thoracic geometry, lung volume, and varying distributions of subcutaneous fat, which obscure the true strength of the cardiac source [7]. In contrast, the cardiac magnetic field permeates biological tissue largely unhindered [8]. Moreover, unlike rigid SQUID dewars, OPM sensors are placed millimeters from the skin. Because magnetic field strength decreases strongly with increasing source-to-sensor distance, the close proximity of OPM sensors may enhance sensitivity to near-field cardiac sources, such as the anteriorly located right ventricle. Therefore, the reduced field strength observed in our ACM cohort may reflect reduced active myocardial source strength; however, larger and age-matched studies are required to determine the extent to which this effect is independent of body habitus, sensor-to-heart distance, and other potential confounders.

Furthermore, these effects were predominantly localized to the lower and right-sided sensor regions (from the patient's perspective, Fig. 6, corresponding to the anatomical position of the right ventricle).

These findings suggest that measuring the heart's magnetic field could supplement the established Task Force criteria and help screen at-risk individuals. At this stage, we cannot yet determine if MCG might also serve as an early disease indicator, especially in diagnostically ambiguous cases; further studies with more participants are needed to establish this.

There are several other issues that have to be considered. In ECG analysis, late potentials are one criterion for ACM. However, the necessary data filtering to remove background noise in MCG recordings also eliminates the low-frequency signals characteristic of late potentials.

Although not in regular use today, vectorcardiography was previously used to diagnose ACM. It had limitations in detecting normal ECG findings and various borderline or abnormal findings such as T-wave inversions and pathological Q waves [10]. Earlier ACM-focused studies reported varying degrees of right ventricular conduction delay, with localized right precordial QRS prolongation described as a highly specific finding in ARVC/ACM subjects [11].

Cardiac MRI is an important imaging modality for detecting structural abnormalities and fatty infiltration in patients with typical ACM. However, fatty infiltration is not always demonstrable on MRI. Correlating VMCG signal attenuation with clinical severity, including MRI findings, would therefore be highly relevant. In the present study, however, the small sample size of nine ACM patients precluded a meaningful statistical correlation analysis. Larger studies comparing MCG-derived parameters with MRI markers of ACM severity are warranted and may help clarify whether MCG provides complementary information on the electrophysiological consequences of the disease.

Overall, little has been reported in the literature about the magnetic field of the heart in subjects with ACM. The biomagnetic pattern we found in patients with ACM is of particular interest and requires further evaluation. The expressivity of the genes within a family is variable. The reduced penetrance is around 60%, meaning that not all carriers will develop ACM. VMCG may help identify early signs in genotype-positive patients who have not yet exhibited any clinical symptoms.

### 4.1 Limitations of the study

There are several limitations that have to be considered.

A key limitation of this study is the low number of cases that could ultimately be included. Patients with implanted ICDs or pacemakers were excluded because their MCG data would be difficult to interpret and potentially non-representative of the patients' intrinsic cardiac disease state due to device-related electromagnetic interference and, where applicable, paced cardiac activation. The strong magnetic fields generated by these devices can introduce electromagnetic interference (EMI) into the MCG recordings. This interference can generate magnetic fields (>50 nT) that exceed the operational dynamic range of the field-nulling algorithm within the optically pumped magnetometers (OPMs) [12]. Moreover, in patients with paced rhythms, the recorded cardiac magnetic signal may partly or predominantly reflect device-driven electrical activation rather than native myocardial conduction, limiting its validity for assessing intrinsic cardiac function. Technical improvements are underway to address these issues. In addition, measurements performed in more strongly shielded environments, such as magnetically shielded rooms (MSRs), require substantially less filtering and may further enhance data quality in future analyses.

Another limitation is the prone position, which was used to maximize signal strength but may be uncomfortable for some patients in routine clinical practice.

In addition, because of the small sample size and repeated sensor-wise testing, all sensor-level findings should be interpreted as exploratory. The principal T-wave convex hull area finding persisted after FDR correction and remained spatially consistent, supporting its prioritization for validation in larger cohorts. Further studies are therefore warranted to confirm these findings and assess their clinical utility.

## 5 Conclusion

Although our data must be interpreted with caution due to the small sample size, we suggest that OPM-based magnetocardiography may represent a promising candidate screening approach for high-risk individuals with ACM, pending validation in larger cohorts. Our optically pumped magnetometer system is cost-effective and patient-friendly, making it suitable for easy integration into clinical routines.

## CRediT authorship contribution statement

**Samuel Friese:** Writing – original draft, Software, Methodology, Conceptualization. **Philipp Wunderl:** Software, Methodology, Investigation, Data curation. **Tobias Jensch:** Data curation. **Lena Wunderl:** Methodology, Data curation. **Cordula M. Wolf:** Writing – review & editing. **Bettina Reich:** Writing – review & editing. **Christian Meierhofer:** Writing – review & editing. **Reinhard Heckel:** Writing – review & editing. **Isabel Diebold:** Writing – review & editing, Data curation. **Eimo Martens:** Writing – review & editing, Data curation. **Dominik Westphal:** Writing – review & editing, Data curation. **Peter Fierlinger:** Writing – review & editing. **Peter Ewert:** Writing – original draft. **Annette Wacker-Gussmann:** Writing – original draft, Supervision, Project administration, Conceptualization.

## Ethics committee approval

The study was reviewed and approved by the ethics committee of the University Hospital, Technical University of Munich, Germany. All patients provided their written informed consent.

## Funding sources

This research did not receive any specific grant from funding agencies in the public, commercial, or not-for-profit sectors.

## Declaration of competing interest

The authors declare that they have no known competing financial interests or personal relationships that could have appeared to influence the work reported in this paper.

## Data Availability

An anonymized data set can be made available upon request. Access is only granted to academic institutions after signing a data sharing agreement.
